# Meta-analysis of PAX1/JAM3 methylation performance in high-risk HPV-positive women

**DOI:** 10.1097/MD.0000000000050757

**Published:** 2026-09-18

**Authors:** Lianxing Qi, Yinfang Wu, Minxia Luo

**Affiliations:** a Department of Laboratory Medicine, Shaoxing Shangyu Maternal and Child Health Hospital, Shaoxing, Zhejiang, China; b Department of Gynecology, Shaoxing Shangyu Maternal and Child Health Hospital, Shaoxing, Zhejiang, China.

**Keywords:** cervical cancer screening, DNA methylation, JAM3, meta-analysis, PAX1

## Abstract

**Background:**

DNA methylation is an emerging biomarker for cervical cancer screening. This study aimed to evaluate the diagnostic performance of paired box gene 1/junctional adhesion molecule 3 (JAM3) dual-gene methylation analysis for detecting high-grade cervical intraepithelial neoplasia and cervical cancer, and to explore its potential utility as a triage biomarker for high-risk human papillomavirus positive women.

**Methods:**

A systematic search was conducted across 5 databases, including PubMed, for studies on PAX1/JAM3 methylation testing in cervical cancer screening. Following quality assessment via the QUADAS-2 tool, statistical analyses were performed using Meta-Disc 1.4 and Stata 17 software.

**Results:**

Eight studies involving 7494 participants were included in the meta-analysis. For diagnosing cervical intraepithelial neoplasia grade 2 and above the pooled sensitivity was 0.81 (95% CI: 0.73–0.88), specificity was 0.95 (95% CI: 0.94–0.96), and the area under the curve was 0.96. For diagnosing cervical intraepithelial neoplasia grade 3 and above the pooled sensitivity was 0.85 (95% CI: 0.75–0.92), specificity was 0.88 (95% CI: 0.86–0.89), and the area under the curve was 0.90.

**Conclusions:**

PAX1/JAM3 dual-gene methylation testing demonstrates high diagnostic accuracy for detecting high-grade cervical intraepithelial neoplasia and cervical cancer, supporting its potential role as a triage biomarker in high-risk human papillomavirus positive women.

## 1. Introduction

Cervical cancer poses a substantial global threat to women’s health. In 2022, there were 661,021 new cases and 348,189 deaths worldwide, ranking it eighth in incidence and ninth in mortality among all cancers, respectively.^[1]^ Many countries have committed to eliminating cervical cancer as a public health goal.^[2]^ A key pillar of this effort is expanding screening coverage among eligible women, and another is ensuring effective triage for abnormal results.

Current cervical cancer screening primarily uses high-risk human papillomavirus (HR-HPV) DNA testing as the initial screen, followed by cytology to triage positive cases.^[3]^ However, this approach has certain limitations: HPV testing has relatively low specificity, and cytological interpretation is inherently subjective. These factors can adversely affect the overall sensitivity and specificity of the screening strategy.^[4–6]^ These drawbacks may result in unnecessary colposcopy referrals and invasive procedures. Therefore, developing improved triage strategies for HR-HPV-positive women represents a critical clinical need.

DNA methylation testing holds significant promise as a novel approach to cervical cancer screening. The 2022 cancer screening review published by the American Cancer Society highlighted a large-sample clinical study conducted by a Chinese team. That study suggested that paired box gene 1/junctional adhesion molecule 3 (JAM3) dual-gene methylation testing has good diagnostic potential in cervical lesion screening.^[7]^ In recent years, several validation studies have been published, but most are single-center and thus have limited generalizability.^[8–10]^ To address this, we conducted a meta-analysis of existing data to evaluate the performance of PAX1/JAM3 methylation testing. Specifically, we focused on its role in cervical cancer screening and risk stratification among HR-HPV-positive women, aiming to provide high-quality evidence for its clinical translation and standardization.

## 2. Methods

### 2.1. Search strategy

We performed a systematic literature search in PubMed, Cochrane Library, Embase, Web of Science, and CNKI. The search covered records from database inception to July 18, 2025. The search strategy combined Medical Subject Headings (MeSH) terms such as “cervical cancer,” “PAX1,” and “JAM3” with free-text keywords. The complete search strings are available in Supplementary Table 1, Supplemental Digital Content 1. The PRISMA 2020 statement was followed in the reporting of this systematic review, which was registered in advance with PROSPERO (CRD420250655385).

### 2.2. Study selection

The inclusion criteria were: English- or Chinese-language studies examining the diagnostic accuracy of dual-gene methylation testing for cervical lesions using PAX1/JAM3. The necessary data to extract the true-positive (TP), true-negative (TN), false-positive (FP), and false-negative (FN) values were available from the included studies. Study population restricted to HR-HPV-positive women; and use of pathological histology as the reference standard for diagnosing cervical lesions.

The exclusion criteria comprised: duplicate publications; studies where outcome data could not be retrieved. Publication types such as reviews, commentaries, or case reports; and animal or basic science studies.

### 2.3. Data extraction

Two investigators independently extracted data using a predefined standardized form. The following information was recorded: first author, publication year, study design, specimen type, detection method, target genes, infection type, sample size, and the number of TP, FP, FN, and TN.

### 2.4. Quality assessment

Two investigators independently assessed the methodological quality of the included studies using the QUADAS-2 tool in RevMan 5.4 (Review Manager, version 5.4, The Cochrane Collaboration). The tool addresses 2 critical aspects risk of bias and concerns regarding applicability within 4 domains: patient selection, index test, reference standard, and flow and timing. Any disagreements between the assessors were resolved through consultation with a third reviewer.

### 2.5. Statistical analysis

We conducted all statistical analyses with Meta-Disc 1.4 (Meta-Disc, version 1.4; developed by Spanish biostatisticians) and Stata 17.0 (Stata, version 17, StataCorp LLC). Heterogeneity: We used Spearman’s correlation coefficient to assess threshold effect; absence was indicated by *P >* .05. Non-threshold heterogeneity was evaluated utilizing Cochran’s *Q* statistic and the *I^2^* index. If heterogeneity was not significant (Cochran’s *Q*-test *P *> .05 and *I^2^* < 50%), a fixed-effects model was used to pool effect sizes; if not, a random-effects model was employed. Pooled estimates included sensitivity, specificity, positive likelihood ratio (PLR), negative likelihood ratio (NLR), and diagnostic odds ratio (DOR). These were presented along with their forest plots, summary receiver operating characteristic (SROC) curve, and the area under the curve (AUC). When significant non-threshold heterogeneity was found, we explored its sources using meta-regression, subgroup analysis, or sensitivity analysis. Sensitivity Analysis: The robustness of the pooled results was evaluated using a leave-one-out approach, whereby each study was sequentially excluded, and Publication Bias: Potential publication bias was assessed using Deeks’ funnel plot asymmetry test.

### 2.6. Ethics statement

As this study is a systematic review and meta-analysis of previously published data, ethical approval and informed consent were not required.

## 3. Results

### 3.1. Literature selection process and results

**Figure 1.**
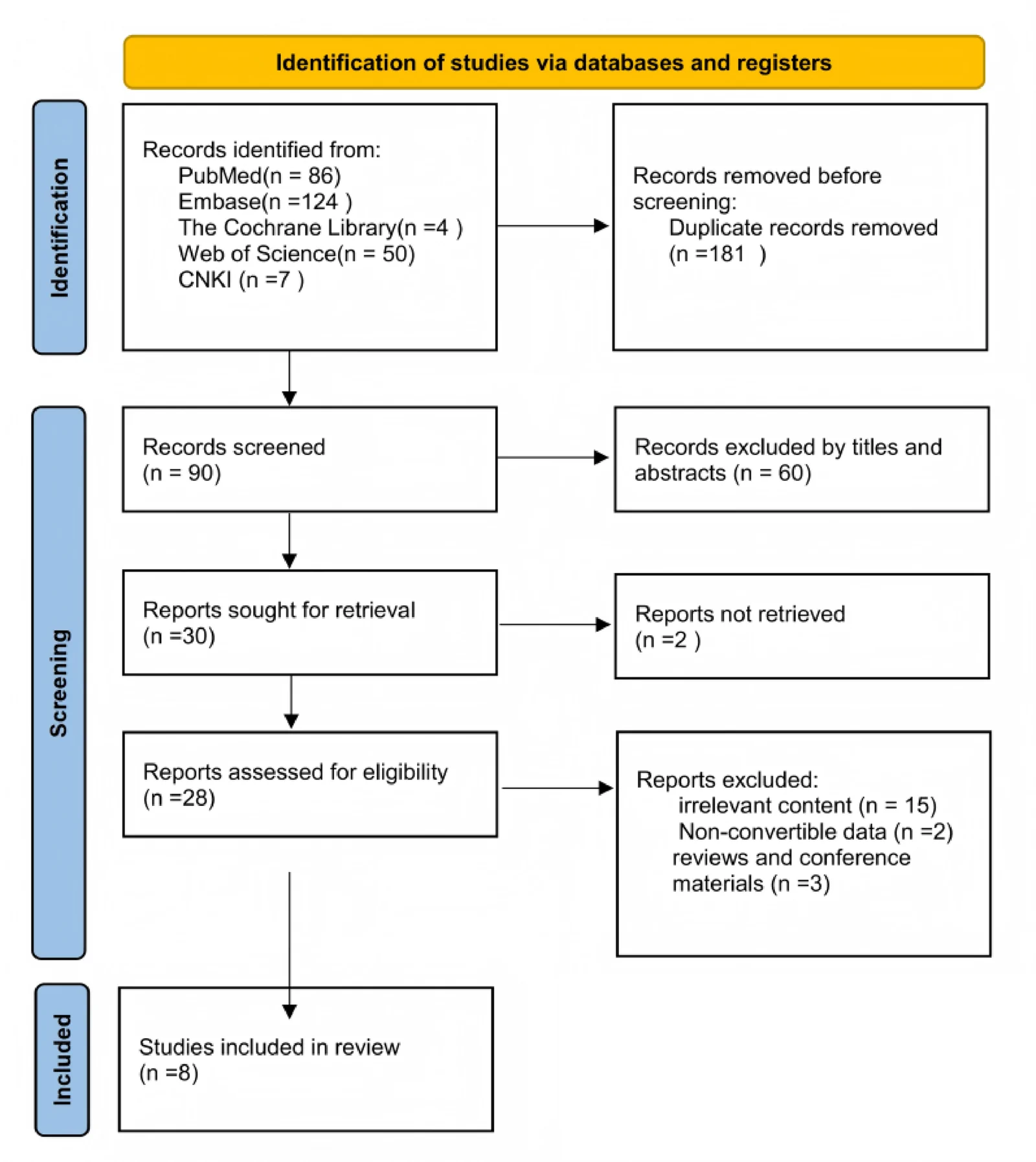
Schematic flow diagram for the selection of included studies.

Initially, 271 records were identified in the electronic databases using the predefined search technique. Deduplication removed 181 records. Subsequent screening of titles and abstracts led to a comprehensive text evaluation for eligibility against the predefined criteria. The final inclusion consisted of 8 studies, encompassing a total of 7494 participants.^[11–18]^ The study selection process and baseline characteristics are presented in Figure 1 and Table 1.

**Table 1:** General characteristics of the included studies.

Author	Publication yr	Study design	Specimen type	Detection method	Target genes	Infection type	Sample size	Number of CIN2+ (%)	Number of CIN3+ (%)	CIN2+ group				CIN3+ group			
										TP	FP	FN	TN	TP	FP	FN	TN
Liang H et al.^[11]^	2024	Cohort	Cervical sample	RT-PCR	PAX1/JAM3	HR-HPV	396	94 (23.7%)	–	87	13	7	289	–	–	–	–
Kong L et al.^[12]^	2025	Prospective multicenter cohort	Cervical sample	RT-PCR	PAX1/JAM3	HR-HPV	1105	-	123 (11.1%)	–	–	–	–	84	102	39	880
Chen X et al.^[13]^	2024	Cohort	Cervical Sample	RT-PCR	PAX1/JAM3	HR-HPV	185	37(20%)	17 (9.2%)	31	7	6	141	15	23	2	145
Shang X et al.^[14]^	2024	Prospective multicenter cohort	Cervical sample	RT-PCR	PAX1/JAM3	HR-HPV	1184	370(31.3%)	202 (17.1%)	259	28	111	786	166	121	36	861
Su H et al.^[15]^	2025	Prospective multicenter cohort	Cervical sample	RT-PCR	PAX1/JAM3	Non-16/18 HR-HPV	643	113(17.6%)	49 (7.6%)	86	26	27	504	44	68	5	526
Chen X et al.^[16]^	2024	Prospective multicenter cohort	Cervical sample	RT-PCR	PAX1/JAM3	Non-16/18 HR-HPV	1851	329(17.8%)	151 (8.2%)	240	83	89	1439	128	195	23	1505
Fei J et al.^[17]^	2024	Cohort	Cervical sample	RT-PCR	PAX1/JAM3	HPV16/18	334	100(29.9%)	53 (15.9%)	89	11	11	223	52	48	1	233
Shang X et al.^[18]^	2025	Prospective cohort	Cervical sample	RT-PCR	PAX1/JAM3	HR-HPV	1796	–	164 (9.1%)	–	–	–	–	122	182	42	1450

CIN2+ = cervical intraepithelial neoplasia grade 2 and above, CIN3+ = cervical intraepithelial neoplasia grade 3 and above, HPV 16/18 = HPV 16 and (or) HPV 18 types, HR-HPV = HPV 16, 18, 31, 33, 35, 39, 45, 51, 52, 56, 58, 59, 66 and 68, JAM3 = junctional adhesion molecule 3, Non-16/18 HR-HPV = HPV 31, 33, 35, 39, 45, 51, 52, 56, 58, 59, 66 and 68, PAX1 = paired box gene 1, RT-PCR = Real-time PCR.

### 3.2. Quality assessment

**Figure 2.**
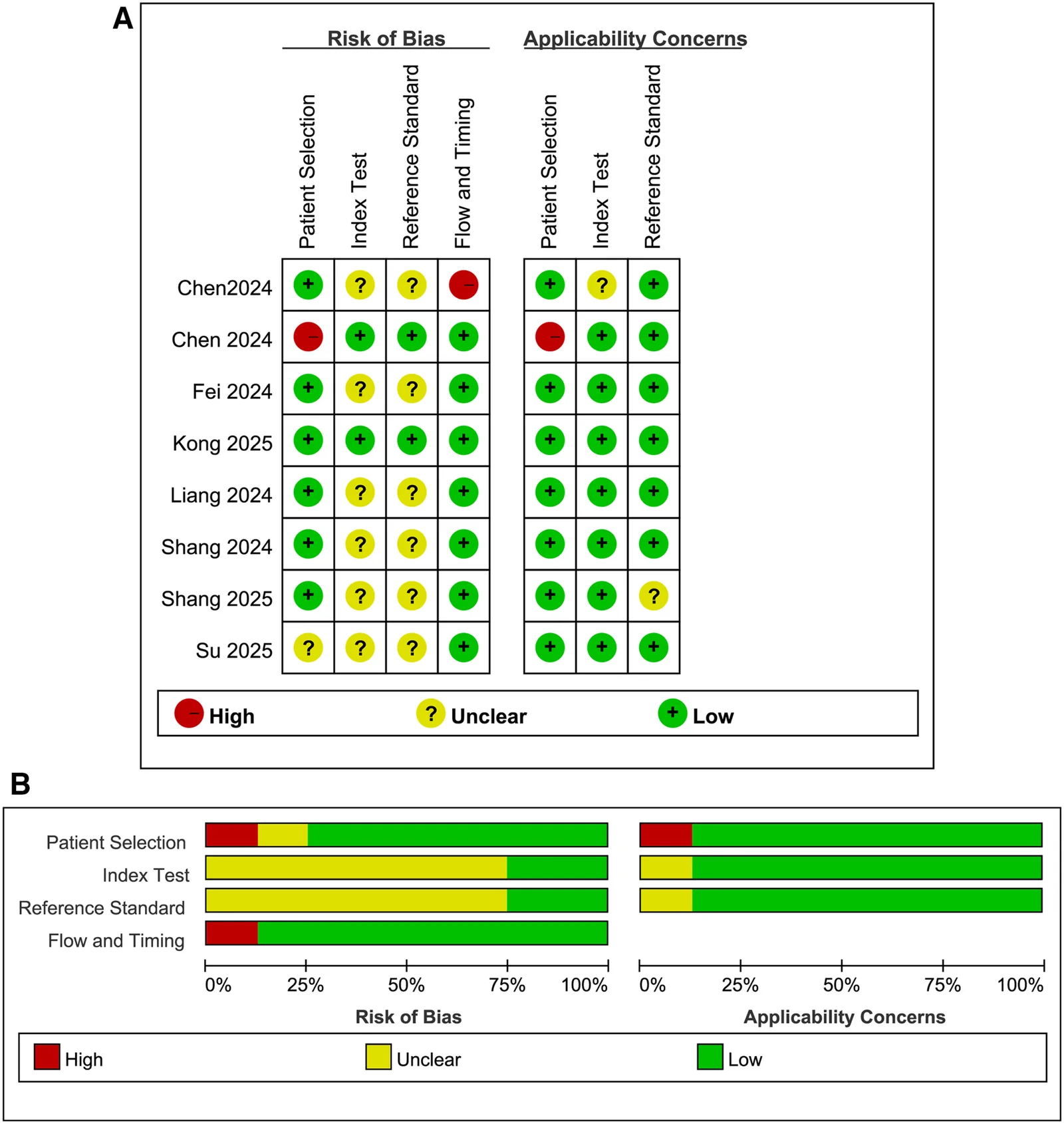
QUADAS-2 risk of bias graph. (A) risk of bias summary graph. (B) risk of bias bar chart. QUADAS-2 = quality assessment of diagnostic accuracy studies 2.

The QUADAS-2 assessment of methodological quality is summarized in Figure 2. Overall, most studies were rated as having a low to moderate risk of bias, indicating relatively high methodological quality. The primary potential sources of bias included: lack of explicit reporting on whether the index test was interpreted blinded to the reference standard results; and insufficient information on whether the reference standard was assessed blinded to the index test results.

### 3.3. Heterogeneity analysis

Diagnostic accuracy data for cervical intraepithelial neoplasia grade 2 and above (CIN2+) and grade 3 and above cervical intraepithelial neoplasia grade 3 and above (CIN3+) were analyzed using Meta-Disc 1.4 software. The Spearman correlation coefficient was -0.143 (*P* = .787) for CIN2+ and 0.750 (*P* = .052) for CIN3+, with both *P*-values exceeding .05. In addition, the SROC curves did not exhibit a characteristic “shoulder-arm” pattern, suggesting the absence of a threshold effect.

In contrast, Cochran’s *Q* test for the DOR revealed significant heterogeneity in both CIN2+ and CIN3 + detection. For CIN2+, Cochran’s *Q* was 17.86 (*P* = .0031) with an *I^2^* of 72.0%; for CIN3+, it was 17.02 (*P *= .0092) with an *I^2^* of 64.8%. Since the *P*-values were ≤0.05 and *I^2^* values exceeded 50% for both endpoints, the observed heterogeneity was considered to arise from non-threshold sources. Therefore, the effect sizes were pooled using a random-effects model.

### 3.4. Meta-analysis results

**Figure 3.**
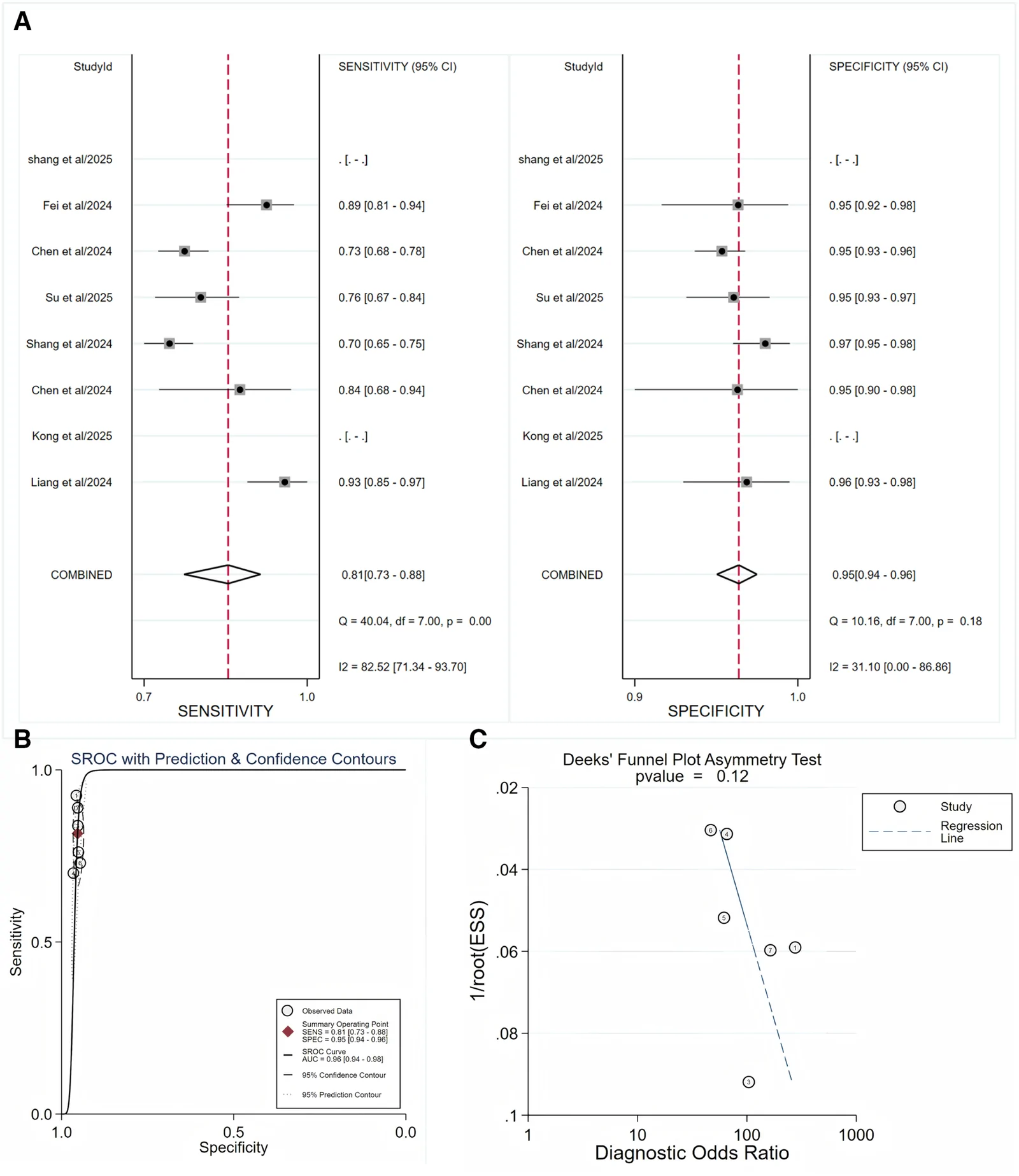
Forest plot for the detection of CIN2+ by PAX1 and JAM3 gene methylation. (A) Diagnostic sensitivity and diagnostic specificity. (B) SROC curve. (C) Deek’s funnel plot assessing the publication bias of included studies. AUC = area under the curve, CIN2+ = cervical intraepithelial neoplasia grade 2 and above, PAX1 = paired box gene 1, JAM3 = junctional adhesion molecule 3, SROC = summary receiver operating characteristic.

We calculated the pooled effect sizes for detecting CIN2+ and CIN3+ with a random-effects model in Stata 17. For CIN2+ detection, the pooled sensitivity was 0.81 (95% CI: 0.73–0.88) and specificity was 0.95 (95% CI: 0.94–0.96) (Fig. 3A). The PLR was 17.5 (95% CI: 14.0–21.7), the NLR was 0.19 (95% CI: 0.13–0.29), and the DOR was 90 (95% CI: 53–154).The SROC curve AUC was 0.96 (95% CI: 0.94–0.98) (Fig. 3B).

**Figure 4.**
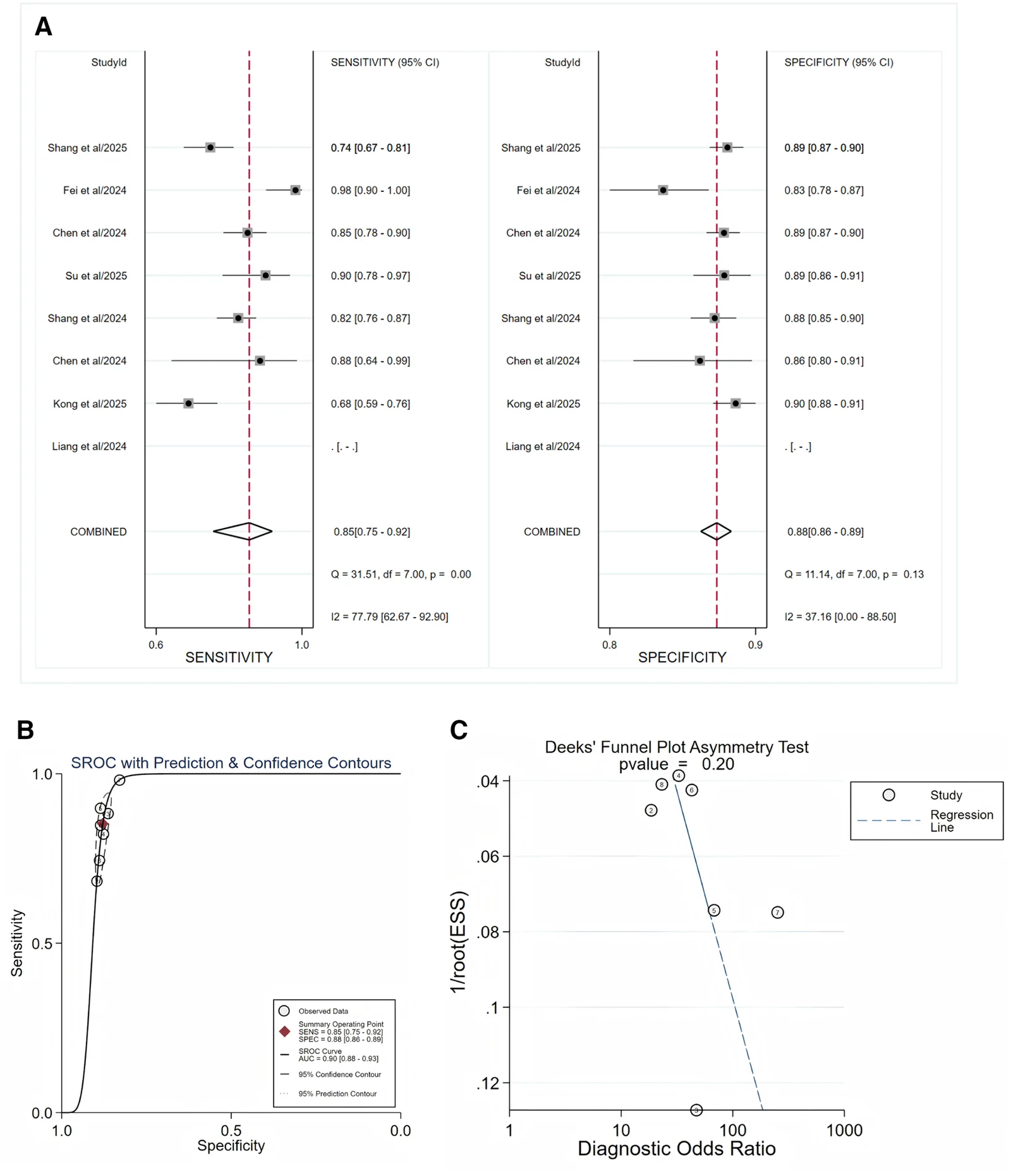
Forest plot for the detection of CIN3+ by PAX1 and JAM3 gene methylation. (A) Diagnostic sensitivity and diagnostic specificity. (B) SROC curve. (C) Deek’s funnel plot assessing the publication bias of included studies. CIN3+ = cervical intraepithelial neoplasia grade 3 and above, PAX1 = paired box gene 1, JAM3 = junctional adhesion molecule 3, SROC = summary receiver operating characteristic.

For CIN3+ detection, the pooled sensitivity was 0.85 (95% CI: 0.75–0.92) and specificity was 0.88 (95% CI: 0.86–0.89) (Fig. 4A).The PLR was 7.0 (95% CI: 6.5–7.6), the NLR was 0.17 (95% CI: 0.10–0.29), and the DOR was 42 (95% CI: 24–73). The SROC curve AUC was 0.90 (95% CI: 0.88–0.93) (Fig. 4B).

### 3.5. Meta-regression and subgroup analysis

**Figure 5.**
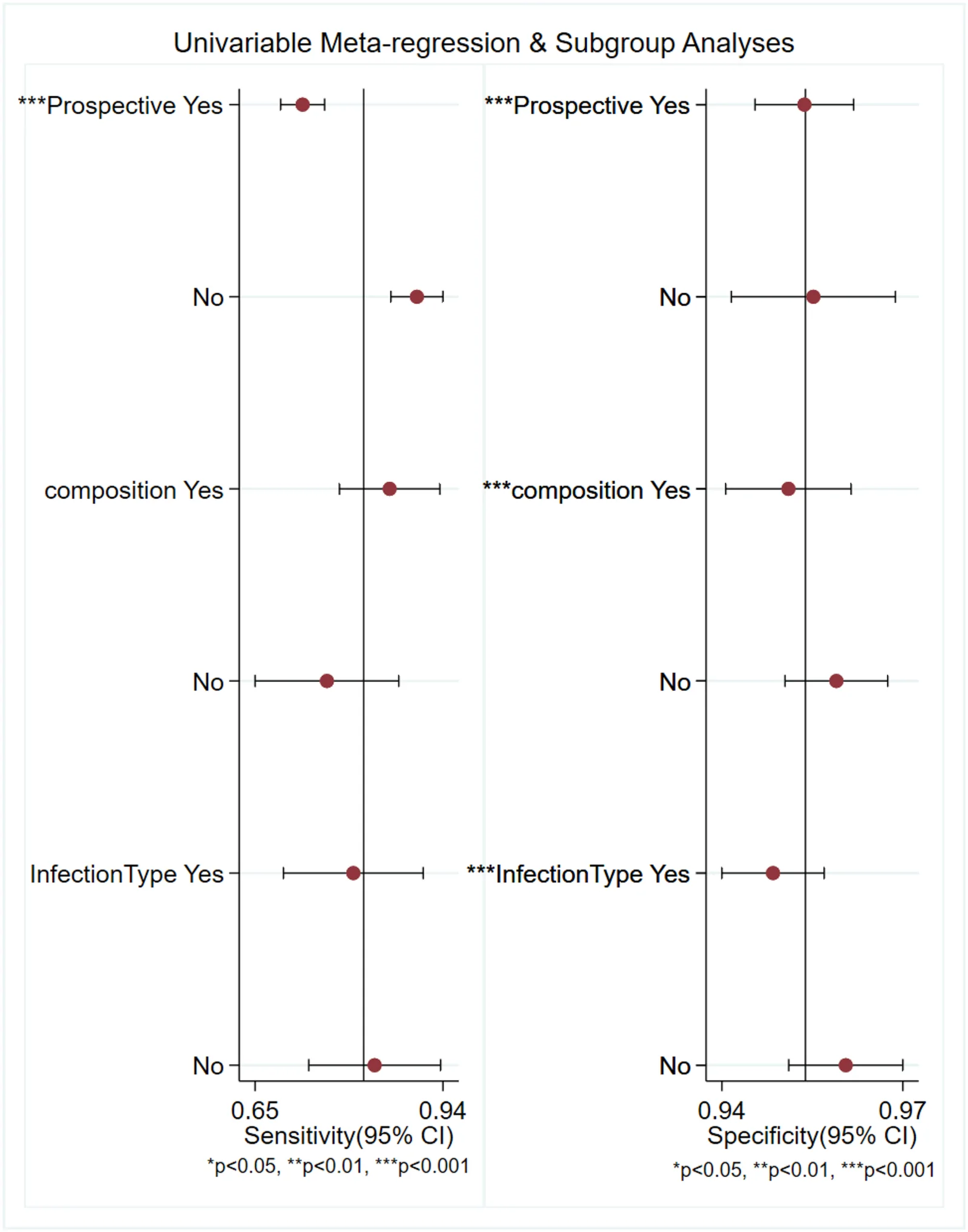
Meta-regression plot of PAX1/JAM3 Methylation testing for diagnosing CIN2+. CIN2+ = cervical intraepithelial neoplasia grade 2 and above, PAX1 = paired box gene 1, JAM3 = junctional adhesion molecule 3.

To explore potential sources of heterogeneity, we performed meta-regression and subgroup analyses on the CIN2+ studies. Covariates, including study design, HR-HPV infection type, and sample composition, were incorporated into the regression model. The analysis identified study design as a key factor contributing to heterogeneity (*P* < .05) (Fig. 5). Subsequent subgroup analysis showed that prospective studies demonstrated higher sensitivity than retrospective studies, while specificity remained identical. Studies with a higher proportion of CIN2+ cases demonstrated higher sensitivity but slightly lower specificity compared to those with a lower CIN2+ prevalence. Additionally, studies defining HR-HPV infection types as encompassing 14 genotypes showed higher diagnostic accuracy than those categorizing infections as “Non-16/18hours-HPV and 16/18hours-HPV” (Table 2).

**Table 2:** Subgroup analysis for the selected studies.

Subgroup	N	Sensitivity (95%*CI*)	Specificity (95%*CI*)	DOR (95%*CI*)	AUC (95%*CI*)
Prodesign					
Retrospective study	3	0.72 (0.69–0.75)	0.95 (0.94–0.96)	53 (41–69)	0.88 (0.85–0.91)
Prospective study	3	0.90 (0.85–0.93)	0.95 (0.94–0.97)	182 (104–317)	0.98 (0.96–0.99)
Sample composition (CIN2+%)					
≤20%	3	0.74 (0.65–0.83)	0.96 (0.94–0.97)	64 (45–93)	0.95 (0.93-0.97)
>20%	3	0.86(0.74–0.93)	0.95(0.94–0.96)	121 (48–307)	0.96 (0.94–0.98)
Infection type					
HR-HPV	3	0.84 (0.73–0.93)	0.96 (0.95–0.97)	124 (96–274)	0.97 (0.95–0.98)
Non-16/18 h-HPV and 16/18 h-HPV	3	0.80 (0.70–0.87)	0.95 (0.94–0.96)	73 (39–135)	0.96 (0.94–0.97)

AUC = area under the curve, CIN2+ = cervical intraepithelial neoplasia grade 2 and above, DOR = diagnostic odds ratio, HPV 16/18 = HPV 16 and (or) HPV 18 types, HR-HPV = HPV 16, 18, 31, 33, 35, 39, 45, 51, 52, 56, 58, 59, 66 and 68, Non-16/18 hour-HPV = HPV 31, 33, 35, 39, 45, 51, 52, 56, 58, 59, 66 and 68.

### 3.6. Sensitivity analysis

To evaluate the stability of our findings, a leave-one-out approach was applied as a sensitivity analysis in Stata 17. This involved sequentially excluding each study to evaluate its influence on the pooled effect estimates. The pooled estimates remained stable across all iterations, indicating the robustness of the meta-analysis. No individual study had a decisive influence on the pooled results.

### 3.7. Publication bias

To assess publication bias, we applied Deeks’ funnel plot asymmetry test. The results indicated no significant asymmetry for either CIN2 + (*P* = .12; Figure 3C) or CIN3+ detection (*P* = .20; Figure 4C), indicating no substantial publication bias (significance threshold: *P* > .05).

## 4. Discussion

Owing to its superior sensitivity, HR-HPV nucleic acid testing is the first-line screening approach for cervical cancer. However, its relatively low specificity and inability to distinguish transient infections can lead to patient anxiety and unnecessary follow-up procedures.^[19]^ Therefore, this study evaluated the diagnostic performance of PAX1/JAM3 methylation testing, a novel biomarker, for cervical cancer screening.

DNA methylation is a key epigenetic modification, and aberrant methylation contributes to tumorigenesis and cancer progression.^[20]^ Methylation-based assays are increasingly used in early cancer screening, disease monitoring, and treatment planning.^[21–23]^ Previous studies on single-gene methylation of PAX1 or JAM3 have demonstrated their potential utility in cervical cancer screening.^[24,25]^ More recently, combined PAX1/JAM3 dual-gene testing has attracted attention, with evidence suggesting that the dual-gene assay outperforms single-gene tests in detection accuracy.^[10,26]^ However, a comprehensive synthesis and evaluation of the available evidence has been lacking. This meta-analysis fills that gap by systematically summarizing the evidence on PAX1/JAM3 dual-gene testing. In addition, all included studies were published within the last 2 years, underscoring the timeliness of this evidence synthesis. A further distinctive feature of this study is its specific focus on HR-HPV-positive populations, evaluating the potential role of PAX1/JAM3 methylation testing in triaging these individuals.

This meta-analysis included 8 studies published within the past 2 years, involving 7494 participants. The pooled results indicated that the PAX1/JAM3 dual-gene methylation assay achieved sensitivities of 0.81 for CIN2+ and 0.85 for CIN3+, with specificities of 0.95 and 0.88, in the same order. These results indicate good diagnostic accuracy, particularly the high specificity of 0.95 for CIN2+, which may help minimize false-positive results and reduce overtreatment. The PLRs were 17.5 for CIN2+ and 7.0 for CIN3+, suggesting that positive results were substantially more likely in diseased than non-diseased individuals. Conversely, the negative NLRs were 0.19 and 0.17, supporting a high ability to rule out disease. The DORs were 90 for CIN2+ and 42 for CIN3+. Furthermore, the areas under the SROC curves were 0.96 for CIN2+ and 0.90 for CIN3+, providing strong evidence for the overall efficacy of the PAX1/JAM3 methylation test. Meta-regression and subgroup analyses for CIN2+ detection identified study design as a significant source of heterogeneity. Subgroup analysis suggested that prospective studies and those with a higher proportion of CIN2+ cases were associated with higher sensitivity, whereas studies defining HR-HPV positivity as encompassing 14 genotypes demonstrated superior diagnostic accuracy.

Based on the findings of this meta-analysis, we propose the following perspectives regarding the clinical implementation of PAX1/JAM3 dual-gene methylation testing. First, methylation testing can be adopted for triage of HR-HPV-positive individuals. For high-risk patients with concurrent HR-HPV positivity and positive methylation results, colposcopy and pathological biopsy can be prioritized to shorten the diagnostic interval for cervical lesions. For HR-HPV-positive women with negative methylation results, unnecessary invasive procedures can be avoided in low‑risk patients after comprehensive physician evaluation. Second, standalone methylation testing may serve as a primary screening tool for cervical cancer. Methylation testing offers the advantages of strong objectivity and ease of standardization. When integrated with self-sampling techniques, it will have broader clinical applicability. Third, methylation testing can be applied in post treatment surveillance and recurrence monitoring. Its noninvasive nature renders it suitable for long-term follow-up of patients after cervical lesion treatment; nevertheless, additional investigations are required to validate the correlation between dynamic methylation changes and disease recurrence for this indication.

The main limitation of this study is that all included studies were conducted in mainland China, which may limit the generalizability of the findings to other ethnic and geographic populations. Furthermore, the limited pool of eligible studies and the presence of significant heterogeneity may affect the robustness of the conclusions. We hope this study will draw international attention to the PAX1/JAM3 methylation assay and encourage further validation across diverse populations and settings. These advancements could promote the broader clinical application of this method, enhancing strategies for both the early detection and tailored treatment of cervical cancer.

## 5. Conclusion

PAX1/JAM3 dual-gene methylation testing demonstrates high diagnostic accuracy for detecting high-grade cervical intraepithelial neoplasia and cervical cancer, supporting its potential role as a triage biomarker in HR-HPV-positive women.

## Author contributions

**Data curation:** Lianxing Qi, Yinfang Wu.

**Formal analysis:** Lianxing Qi, Yinfang Wu.

**Funding acquisition:** Lianxing Qi.

**Investigation:** Lianxing Qi.

**Methodology:** Lianxing Qi.

**Project administration:** Lianxing Qi.

**Resources:** Lianxing Qi.

**Software:** Lianxing Qi.

**Supervision:** Lianxing Qi.

**Validation:** Lianxing Qi.

**Visualization:** Lianxing Qi.

**Conceptualization:** Minxia Luo.

**Writing – original draft:** Lianxing Qi

**Writing – review & editing:** Minxia Luo.


