# Supplementary material for: Meta-analysis of PAX1/JAM3 methylation performance in high-risk HPV-positive women
Source: Medicine (Baltimore). 2026 Sep 18;105(38):e50757. doi: 10.1097/MD.0000000000050757 (PMC13592966; doi:10.1097/MD.0000000000050757)
Supplement: Supplementary file 1 [file medi-105-e50757-s001.docx]

**Supplementary Table 1. Search Strategy**

|  | Search No. | Search Strategy | Final Combined Strategy |
| --- | --- | --- | --- |
| PubMed | #1 | (("DNA Methylation"[Mesh] OR methylat*[Title/Abstract]) AND ("PAX1"[Title/Abstract] OR "JAM3"[Title/Abstract] OR "Jam3"[Title/Abstract])) | #1 AND #2 AND #3 |
|  | #2 | ("Uterine Cervical Neoplasms"[Mesh] OR "cervical cancer"[Title/Abstract] OR "CIN"[Title/Abstract] OR "HSIL"[Title/Abstract] OR "HPV"[Title/Abstract]) |  |
|  | #3 | ("Mass Screening"[Mesh] OR "Early Detection of Cancer"[Mesh] OR screen*[Title/Abstract] OR triage[Title/Abstract] OR "diagnostic accuracy"[Title/Abstract] OR "sensitivity and specificity"[Mesh]) |  |
| Embase | #1 | ('DNA methylation'/exp OR 'methylation'/exp OR methylat*:ti,ab,kw) AND ('PAX1 gene'/exp OR 'JAM3 gene'/exp OR 'PAX1':ti,ab,kw OR 'JAM3':ti,ab,kw) | #1 AND #2 AND #3 |
|  | #2 | ('Uterine cervix cancer'/exp OR 'Cervix intraepithelial neoplasia'/exp OR 'cervix cancer':ti,ab,kw OR 'cervical cancer':ti,ab,kw OR 'CIN':ti,ab,kw OR 'HSIL':ti,ab,kw OR 'Human papillomavirus'/exp OR 'HPV':ti,ab,kw) |  |
|  | #3 | ('Cancer screening'/exp OR 'Early cancer diagnosis'/exp OR screen*:ti,ab,kw OR triage:ti,ab,kw OR 'diagnostic accuracy':ti,ab,kw OR 'sensitivity and specificity'/exp OR 'sensitivity':ti,ab,kw OR 'specificity':ti,ab,kw) |  |
| Cochrane Library | #1 | ((DNA NEXT methylation) OR methylat*) AND (PAX1 OR JAM3) | #1 AND #2 AND #3 |
|  | #2 | ("uterine cervical neoplasms" OR "cervical cancer" OR CIN OR HSIL OR HPV) |  |
|  | #3 | ("mass screening" OR "early detection of cancer" OR screen* OR triage OR "diagnostic accuracy" OR "sensitivity and specificity") |  |
| Web of Science | #1 | (("DNA Methylation" OR methylat*) AND ("PAX1" OR "JAM3" OR "Jam3")) | #1 AND #2 AND #3 |
|  | #2 | ("Uterine Cervical Neoplasms" OR "cervical cancer" OR "CIN" OR "HSIL" OR "HPV") |  |
|  | #3 | ("Mass Screening" OR "Early Detection of Cancer" OR screen* OR triage OR "diagnostic accuracy" OR "sensitivity and specificity") |  |
| CNKI | #1 | (("DNA甲基化"OR "甲基化" AND ("PAX1" OR "JAM3" OR "Jam3") | #1 AND #2 AND #3 |
|  | #2 | ("宫颈肿瘤" OR "宫颈癌" OR "CIN" OR "HSIL" OR "HPV") |  |
|  | #3 | ("大规模筛查" OR "肿瘤早期诊断" OR "筛查" OR "分流诊断" OR "诊断准确性" OR "敏感性与特异性") |  |
